# Time‐dependent bladder activity changes in streptozotocin‐induced female diabetic rats

**DOI:** 10.14814/phy2.70220

**Published:** 2025-02-20

**Authors:** Keiichiro Izumi, Tadanobu Chuyo Kamijo, Takuma Oshiro, Ryu Kimura, Asuka Ashikari, Masahiro Kurobe, Takahiro Akimoto, Minoru Miyazato

**Affiliations:** ^1^ Department of Systems Physiology, Graduate School of Medicine University of the Ryukyus Okinawa Japan; ^2^ Department of Urology, Graduate School of Medicine University of the Ryukyus Okinawa Japan

**Keywords:** bladder activity, bladder contraction, carbachol, detrusor underactivity, overactive bladder

## Abstract

This study aimed to investigate the long‐term physiological and morphological changes in the bladders of diabetic rats. Sixty‐nine female Sprague–Dawley rats were divided into a control and six diabetic (3 days and 2, 4, 8, 12, and 24 weeks after induction of type 1 diabetes) groups. Metabolic cages and cystometry were used to evaluate bladder function. Bladder contractility was assessed using an organ bath test, and Masson's trichrome staining was performed. In the metabolic cage study, the urination frequency during the dark period significantly increased in the early stages at 3 days of diabetes (*p* < 0.05). The voiding interval significantly increased (*p* < 0.05) at 8–12 weeks of diabetes, while the residual urine volume and voiding efficiency worsened at 24 weeks. In the organ bath study, the dose–response curve of carbachol for median effective concentration did not change; however, the bladder contractile force was enhanced at 8 weeks (*p* = 0.028). Histological analysis revealed increased fibrosis at 4 weeks of diabetes. Diabetic bladder dysfunction is characterized by storage and voiding bladder activity changes in the early stages that induces urinary frequency and reduced voiding efficiency in the late phase; this turning point occurs at 8 weeks after diabetes.

## Graphical abstract

Diabetic bladder dysfunction is characterized by storage and voiding bladder activity changes in the early stages that induces overactive bladder and underactive bladder in the late phase; this turning point occurs at 8 weeks after diabetes.

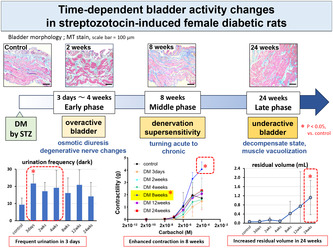

## 1 INTRODUCTION

Diabetic lower urinary tract dysfunction (LUTD) is one of the most common complications of diabetes mellitus (DM), affecting 25%–85% of people with DM (Daneshgari et al., 2009; Ioanid et al., 1981). DM is associated with an increased risk of urinary incontinence (Brown et al., 2003; Diokno et al., 1990) as well as urinary tract infection and renal failure; therefore, it may be ultimately life‐threatening and its prevention is important to maintain daily activities. However, the pathology of diabetic LUTD is diverse, and its treatment is extremely complex (Liu & Daneshgari, 2014).

Normal voiding relies on the coordinated action between the bladder and urethral smooth muscle/external urethral sphincter, which is regulated by the parasympathetic, sympathetic, and pudendal nerves, respectively (Fowler et al., 2008). The typical symptoms of diabetic LUTD include decreased bladder sensation and increased bladder capacity, resulting in impaired urine output and increased residual urine volume (RUV) (Cao et al., 2019; Frimodt‐Møller, 1980). In the early stages, polyuria causes bladder enlargement and increased contractility, and the disease progresses with accumulation of toxic metabolites (Daneshgari, Liu, & Imrey, 2006). This leads to decreased urinary function and dysfunction of the urinary bladder muscles, resulting in an atonic bladder (i.e., underactive bladder) in the final stages (Cao et al., 2019, Frimodt‐Møller, 1980). However, the natural history of diabetic LUTD, particularly the turning point from an early compensatory phase to a decompensatory phase, remains unclear.

Streptozotocin (STZ) has been widely used in type I DM models (Gvazava et al., 2018; Szkudelski, 2001). Using this model, physiological bladder activity changes in vivo have been reported at 9–12 weeks (Daneshgari, Huang, et al., 2006; Daneshgari, Liu, & Imrey, 2006), and in vitro at 16 weeks (Masuda et al., 2020). In addition, morphological changes in the bladder, such as bladder hypertrophy, were observed at a relatively early stage of 5 weeks (Pitre et al., 2002). However, it is still unknown whether physiological in vivo, in vitro bladder activity, or morphological changes are signs of changes from the compensatory phase to the decompensatory phase.

Therefore, this study aimed to elucidate the changes in physiological bladder activity and morphological changes at extended long‐term over 24 weeks in replication STZ‐induced diabetic models. Accordingly, we examined the voiding behavior in the metabolic cage, cystometry, in vitro bladder contractility using an organ bath setup, and bladder histology.

## 2 MATERIALS AND METHODS

### 2.1 Animals

This experiment used 69 8‐week‐old female Sprague–Dawley rats (Kyudo, Saga, Japan) at the animal facility over 7 days, that were divided into seven groups (a control and six DM subgroups). The animals were kept in cages for at least 7 days before the start of the experiment and had free access to standard food (Nippon Clare, Tokyo, Japan) and water. All animals were housed in an animal housing room at controlled temperature (22 ± 0.5°C) and humidity (50% ± 10%) under artificial lighting with a 12 h light/dark cycle.

All experimental procedures were performed using a protocol (A2021078) approved by the Institutional Animal Care and Use Committee of the University of the Ryukyus in compliance with the ARRIVE guidelines (https://arriveguidelines.org/). All efforts were made to minimize suffering and the number of rats used in the experiment.

### 2.2 Type 1 DM model

Type I DM was induced via intraperitoneal injection of 65 mg/kg STZ (Sigma‐Aldrich Co., LLC, MO, USA, Cat No. S0130) dissolved in saline under isoflurane inhalation anesthesia. The animals were not subjected to a specific fasting period. DM was defined as a blood glucose level of at least 250 mg/dL at any time during blood glucose measurement, 3 days after STZ administration.

### 2.3 Metabolic cage

The excretory behavior of rats in seven groups (*n* = 6–8 per group) was evaluated. Rats were housed in metabolic cages (Metabolica™; Sugiyama‐gen Co. Ltd., Tokyo, Japan) in a measurement room with controlled temperature (23 ± 1°C) and humidity (40% ± 5%) and were provided standard rat food and water ad libitum. Urine output was continuously measured for 24 h using a PowerLab™ data acquisition system (ADInstruments, New South Wales, Australia) and a customized GX‐600R electronic scale (A&D Co. Ltd., Tokyo, Japan). Individual urine volumes and urination frequencies were determined during the dark (12 h, from 8 p.m. to 8 a.m.) and light (12 h, from 8 am to 8 pm) periods and throughout the 24 h period. All rats were subjected to cystometrography (CMG).

### 2.4 CMG

CMG was carried out for all seven groups (*n* = 7 in control; *n* = 6–8 in DM groups at 3 days and 2, 4, 8, 12, and 24 weeks after STZ administration) were used in each group. Under isoflurane inhalation anesthesia, a laparotomy was performed, the bilateral ureters were ligated and dissected, and a polyethylene catheter (Intramedic PE‐50; Becton, Dickinson and Company, NJ, USA) was inserted into the upper bladder to form a vesicostomy. After switching to urethane anesthesia (0.4 g/kg administered intraperitoneally and 0.8 g/kg administered subcutaneously; Sigma‐Aldrich Co.), continuous CMG was performed via intravesical catheter connected to a pressure transducer and an infusion pump through a three‐way stopcock. Physiological saline at room temperature was infused at 3 mL/h, and each parameter related to urination was recorded using the digital PowerLab system™ (ADInstruments, Pty. Ltd.) mentioned above. Baseline pressure, pressure threshold, maximum contraction pressure, duration of bladder contraction, intercontraction interval (ICI), and number of nonvoiding contractions (NVCs) were evaluated when rhythmic bladder contractions stabilized for at least 1 h. Saline volume from the urethral meatus was collected and measured as the voided volume (VV). After a final voiding, the intravesical catheter was disconnected, and then the RUV was measured by withdrawing intravesical fluid through the catheter under gravity. Voiding efficiency (%) was calculated as follows: VV/ (VV + RUV) × 100. At the end of the experiment, the rats were euthanized via intraperitoneal administration of high‐dose pentobarbital (100 mg/kg) without awakening from urethane anesthesia for weighing or bladder morphology.

### 2.5 Organ bath studies

The rats were laparotomized, and the bladder was removed by cutting at the level of the internal urethral orifice and weighed. A horizontal incision was made in the bladder body to create a ring‐shaped section 2 mm wide in seven groups (*n* = 6 in control; *n* = 5–8 in DM). This section was hung onto an L‐shaped hook in a 5 mL tissue bath chamber in an organ bath (Model MTOB‐2A; Physio‐Tech Co., Ltd., Tokyo, Japan), and the chamber was filled with Krebs solution maintained at 37°C and continuously bubbled with 95% O_2_ and 5% CO_2_. One of the bladder rings was fixed to an L‐shaped hook connected to a movable unit to allow passive tension adjustment, and isometric contractions were recorded using the digital PowerLab system used for bladder measurements. After attachment, the ring was stretched and stabilized to a passive tension of 2 g. Contraction was induced using 100 mM KCl (FUJIFILM Wako Pure Chemical Corporation, Osaka, Japan), and the contractile force was measured. A continuous dose of carbachol solution (Sigma‐Aldrich Co., Cat No. PHR1511) at varying concentrations (2 × 10^−8^, 2 × 10^−7^, 2 × 10^−6^, 2 × 10^−5^, and 2 × 10^−4^ M) was administered, and the contractile force of the ring sections was measured. Following this, cumulative concentration‐response curves to %maximal response of carbachol were constructed, and the correction factors for the median effective concentration (EC_50_) were compared between the groups.

### 2.6 Bladder morphology

To identify histological changes in the bladder associated with diabetes over time, bladders were removed from DM rats and controls (*n* = 4 per group). Masson's trichrome staining (ScyTek Laboratories Inc. Lcc, UT, USA, Cat No. TRM‐1‐1FU) was performed according to the standard protocol described below. The explanted bladders were fixed in 10% neutral buffered formalin, and 5 μm‐thick sections were cut with a microtome from the paraffin‐embedded specimens and attached to gelatin‐coated slides. The tissue sections were deparaffinized using xylene and ethanol, followed by staining with Hansen's iron hematoxylin for 5 min. The sections were washed with tap water for 5 min and then stained with Biebrich scarlet acid fuchsin (ScyTek Laboratories Inc., Cat No. SCY‐BSU‐125) for 10 min. The nuclei were stained with light green (ScyTek Laboratories Inc. Lcc, Cat No. SCY‐LGA‐500) instead of methylene blue for 10 min to avoid confusion between blue staining of the nuclei and collagen staining. Nuclei were stained black or blue, cytoplasm, muscles, and erythrocytes red, and collagen fibers green. Finally, after dehydration in a graded ethanol series, the tissue sections were permeabilized with xylene and mounted with coverslips using Permount (FALMA Co., Ltd., Tokyo, Japan). The stained slices were photographed using an optical microscope (ECLIPSE Ci; Nikon Co., Tokyo, Japan). Three randomly selected high‐power fields (×20 objective) surrounding the muscle layer in the bladder were analyzed to quantify the fibrotic areas and eliminate potential submucosal collagenous tissue. The ratios of integrated fibrosis/muscle area in each section were calculated using FIJI ImageJ (ver. ImageJ 1.53q, Java 1.8.0_322 [64 bit], https://imagej.net/Fiji/Download), and the average of the values was obtained.

### 2.7 Statistical analysis

GraphPad Prism (version 8.4.3; GraphPad Software Inc., CA, USA) was used for drawing Figure 2. Data are expressed as the mean ± standard deviation (SD). For nonparametric variables, Steel's multiple comparison test in accordance with the results of the Kruskal–Wallis test was carried out using JMP Pro (version 17.2.0; SAS Institute, NC, USA). Statistical significance was set at *p* < 0.05.

## 3 RESULTS

### 3.1 General characteristics

Table 1 and Table S1 list the body and bladder weights and blood glucose level of rats (*n* = 6 for each group). The body weight was significantly higher at 4–24 weeks of DM compared with the control (215.7 ± 12.77 g) by Steel's multiple comparison test. The bladder weight was significantly higher at 12 weeks of DM compared with the control (0.127 ± 0.021 g). However, no differences were observed in bladder weight/body weight ratios between the groups. Blood glucose levels were significantly elevated at 3 days to 24 weeks of DM compared with the control (191.7 ± 23.91 mg/dL).

**TABLE 1:** General characteristics between groups.

	Control (*n* = 6)	3 days (*n* = 6)	2 weeks (*n* = 6)	4 weeks (*n* = 6)	8 weeks (*n* = 6)	12 weeks (*n* = 6)	24 weeks (*n* = 6)
Body weight (g)	215.7 ± 12.77	214.7 ± 38.47	240.5 ± 12.58	285.5 ± 28.15^a^	299.6 ± 18.49^a^	315.0 ± 8.270^a^	305.2 ± 28.10^a^
Bladder weight (g)	0.127 ± 0.021	0.114 ± 0.015	0.161 ± 0.013	0.188 ± 0.053	0.171 ± 0.039	0.294 ± 0.121^a^	0.266 ± 0.092
Bladder/body (×10^3^)	0.587 ± 0.096	0.539 ± 0.066	0.672 ± 0.082	0.651 ± 0.141	0.575 ± 0.140	0.933 ± 0.386	0.889 ± 0.341
Blood glucose (mg/dL)	191.7 ± 23.91	454.5 ± 205.5^a^	751.7 ± 208.7^a^	453.8 ± 180.8^a^	560.7 ± 191.1^a^	670.8 ± 126.1^a^	692.3 ± 113.4^a^

^a^ *p* < 0.05 versus control (Steel's multiple comparison test).

### 3.2 Metabolic cage

Table 2 and Table S2 present the changes in water consumption and urination of diabetic rats (control: *n* = 6; DM at 3 days: *n* = 6; 2 weeks: *n* = 6; 4 weeks; *n* = 6; 8 weeks: *n* = 8; 12 weeks: *n* = 8; 24 weeks: *n* = 6). Daily water consumption significantly increased from 38.7 ± 4.50 g in the control group to 55.1 ± 8.72 g at 2 weeks of DM (*p* = 0.025), while no differences were observed between the other groups by Steel's multiple comparison test. The frequency of urination during the light period did not differ between the groups. In contrast, the frequency of urination during the dark period significantly increased at 3 days of DM (*p* = 0.039) compared with the control (9.33 ± 4.23). Meanwhile, the frequency of urination in a 24 h period did not change between groups. The total 24 h urine volume increased significantly at 4 weeks of DM compared with the control (15.05 ± 3.52 mL), in addition, the ratio of urine to water intake was significantly increased (*p < 0.05*) at 3 days, 4, 8, and 24 weeks of DM. The volume of urine output per voiding was similar in all groups.

**TABLE 2:** Metabolic cage parameters in the different groups.

	Control (*n* = 6)	3 days (*n* = 6)	2 weeks (*n* = 6)	4 weeks (*n* = 6)	8 weeks (*n* = 8)	12 weeks (*n* = 8)	24 weeks (*n* = 6)
Water intake (g)	38.73 ± 4.50	43.33 ± 15.8	55.13 ± 8.72^a^	40.37 ± 12.29	38.48 ± 20.20	58.17 ± 29.43	40.96 ± 58.06
Urinary fr. (light)	8.67 ± 4.76	10.83 ± 1.60	10.17 ± 2.14	9.33 ± 1.63	7.88 ± 3.91	7.50 ± 2.56	10.00 ± 3.35
Urinary fr. (dark)	9.33 ± 4.23	21.67 ± 5.79^a^	17.17 ± 5.56	19.17 ± 11.16	16.13 ± 9.38	20.88 ± 9.38	14.17 ± 8.89
Urinary fr. (total)	18.00 ± 7.48	32.50 ± 7.12	27.33 ± 6.71	28.50 ± 10.41	24.00 ± 9.47	28.38 ± 10.82	24.17 ± 12.02
Urinary vol. (mL/day)	15.05 ± 3.52	25.49 ± 8.18	25.14 ± 7.02	31.77 ± 21.50^a^	32.02 ± 23.81	37.00 ± 34.70	42.68 ± 51.30
Urinary vol. (mL/time)	1.00 ± 0.67	0.77 ± 0.04	0.93 ± 0.19	1.05 ± 0.26	1.26 ± 0.58	1.15 ± 0.57	1.42 ± 0.94
Urinary vol./water intake	0.39 ± 0.06	0.60 ± 0.06^a^	0.46 ± 0.11	0.74 ± 0.22^a^	0.79 ± 0.23^a^	0.59 ± 0.22	9.68 ± 20.64^a^

*Note*: Frequency; vol, volume.

^a^ *p* < 0.05 versus control (Steel's multiple comparison test).

### 3.3 CMG

**FIGURE 1:**
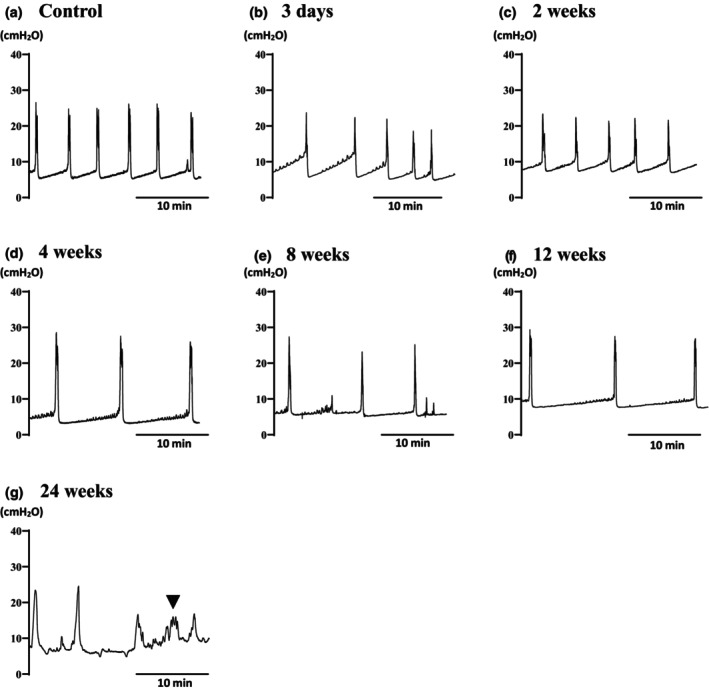
Representative images of cystometry in the control (a, *n* = 7) and 3 days and 2, 4, 8, 12, and 24 weeks after diabetes induction (b–g, *n* = 6–8 each). The voiding interval was longer at 8–12 weeks of diabetes (e, f) compared with the control. Voiding contractions were reduced, and a nonvoiding contraction (▼) was shown 24 weeks after diabetes (g). All scale bars indicate 10 min.

Figure 1 shows representative images of CMG in each group, and Table 3 and Table S3 present the values of each parameter obtained in CMG (control: *n* = 7; DM at 3 days: *n* = 6; 2 weeks: *n* = 6; 4 weeks; *n* = 6; 8 weeks: *n* = 6; 12 weeks: *n* = 8; 24 weeks: *n* = 6). In the control rats, maximum contraction pressure, duration of bladder contraction, and VV were 27.53 ± 9.23 cmH_2_O, 27.5 ± 7.15 s, and 0.234 ± 0.096 mL, respectively. The bladder baseline pressure, bladder contraction threshold, and maximal contraction pressures did not differ between the groups. Compared with the control group (222.1 ± 71.6 s), the voiding interval was significantly prolonged (*p* < 0.05) at 8–12 weeks of DM but was shortened at 24 weeks. VV significantly increased (*p* < 0.05) at 12 weeks but returned to control levels at 24 weeks (0.217 ± 0.142 mL). RUV significantly increased (*p* < 0.05) at 24 weeks compared with the control (0.063 ± 0.065 mL), whereas voiding efficiency significantly decreased (*p* < 0.05) at 24 weeks compared with the control (82.3% ± 16.6%). The duration of bladder contraction and the NVCs did not differ between the groups. Statistical significance was determined by Steel's multiple comparison test.

**TABLE 3:** Cystometric parameters in the different groups.

	Control (*n* = 7)	3 days (*n* = 6)	2 weeks (*n* = 6)	4 weeks (*n* = 6)	8 weeks (*n* = 6)	12 weeks (*n* = 8)	24 weeks (*n* = 6)
BP (cmH_2_O)	8.45 ± 2.21	7.03 ± 2.56	6.99 ± 2.11	7.69 ± 2.26	7.11 ± 4.93	6.70 ± 3.19	5.06 ± 1.38
TP (cmH_2_O)	11.20 ± 2.94	9.77 ± 1.94	9.10 ± 1.72	10.4 ± 2.68	9.20 ± 5.64	10.5 ± 5.59	8.93 ± 2.98
MCP (cmH_2_O)	27.53 ± 9.23	22.59 ± 4.92	22.56 ± 3.80	26.19 ± 6.04	27.02 ± 7.57	32.50 ± 10.34	20.71 ± 2.82
ICI (s)	222.1 ± 71.61	312.2 ± 267.4	227.5 ± 124.3	449.8 ± 266.2	460.5 ± 150.4^a^	673.5 ± 326.1^a^	591.2 ± 568.5
NVCs (times/hour)	0.43 ± 1.13	1.33 ± 1.75	1.33 ± 1.21	0.83 ± 1.69	1.33 ± 1.63	0.63 ± 0.92	0.67 ± 0.816
DBC (s)	27.5 ± 7.15	28.4 ± 4.59	29.7 ± 5.89	34.6 ± 13.3	26.4 ± 3.24	27.4 ± 5.52	44.1 ± 8.68
VV (mL)	0.234 ± 0.096	0.223 ± 0.174	0.315 ± 0.201	0.501 ± 0.243	0.345 ± 0.108	0.506 ± 1.500^a^	0.217 ± 0.142
RUV (mL)	0.063 ± 0.065	0.068 ± 0.078	0.143 ± 0.117	0.090 ± 0.057	0.423 ± 0.535	0.744 ± 1.511	1.122 ± 0.753^a^
VE (%)	82.3 ± 16.6	73.3 ± 29.4	72.1 ± 25.2	81.1 ± 16.2	60.7 ± 26.4	69.0 ± 30.4	20.7 ± 12.3^a^

Abbreviations: BP, basal pressure; DBC, duration of bladder contraction; ICI, intercontraction interval; MCP, maximum contraction pressure; NVCs, nonvoiding contraction; RUV, residual urine volume; TP, threshold pressure; VE, voiding efficiency; VV, voiding volume.

^a^ *p* < 0.05 versus control (Steel's multiple comparison test).

### 3.4 Organ bath studies

**FIGURE 2:**
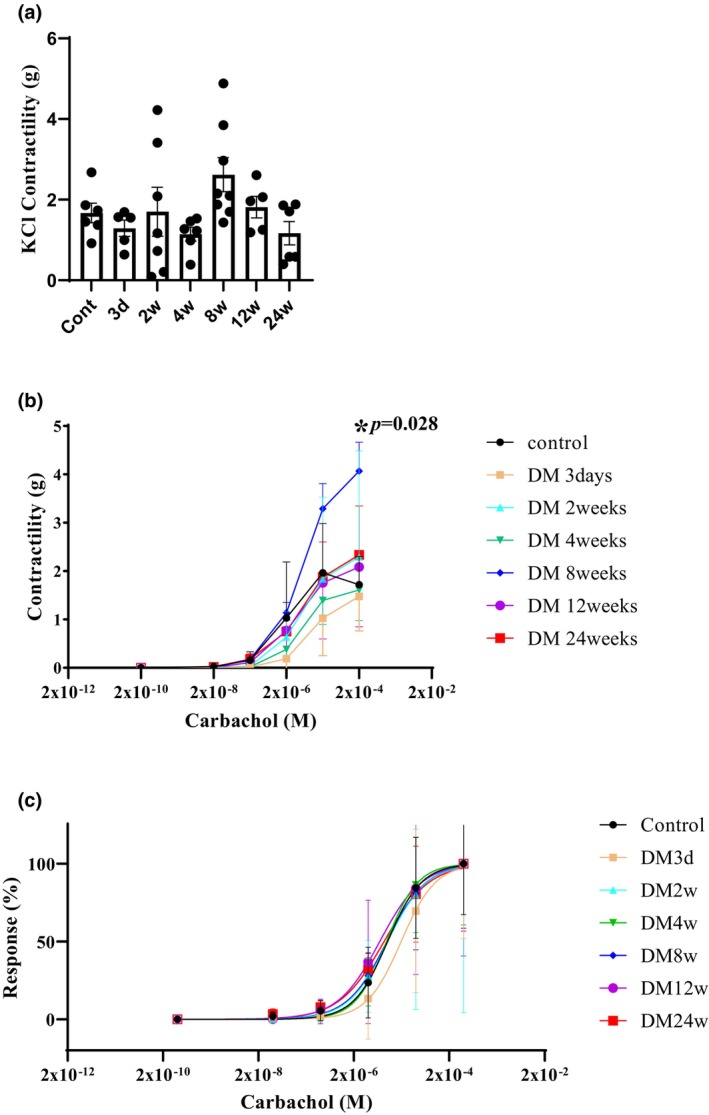
A 100 mM KCl (a) and cumulative carbachol stimulation (2 × 10^−8^, 2 × 10^−7^, 2 × 10^−6^, 2 × 10^−5^, and 2 × 10^−4^ M) to bladder contractile force (b) and dose–response curve (c) in the control (*n* = 6 strips) and 3 days (*n* = 5 strips), 2 weeks (*n* = 7 strips), 4 weeks (*n* = 6 strips), 8 weeks (*n* = 8 strips), 12 (*n* = 6 strips), and 24 weeks (*n* = 6 strips) after diabetes. (a) Bladder contractile force in response to 100 mM KCl stimulation did not differ between groups. (b) Bladder contractile force in response to cumulative carbachol stimulation was significantly enhanced at 8 weeks of DM. (c) The dose–response curve showed no difference depending on the concentration of carbachol (EC_50_ = 2 × 10^−5.75^ M). Data are expressed as the mean ± standard deviation. Statistical significance was determined by Steel's multiple comparison test. *p < 0.05.

The maximal contraction of the bladder ring in response to KCl stimulation of DM groups (*n* = 5–8 strips) did not differ compared with the control (*n* = 6 strips, 1.67 ± 0.59 g) (Figure 2a). In the control bladders, bladder contractions in response to carbachol were observed at 0.026 ± 0.044 g at 2 × 10^−8^, 0.158 ± 0.174 g at 2 × 10^−7^, 1.031 ± 1.159 g at 2 × 10^−6^, 1.967 ± 1.019 g at 2 × 10^−5^, and 1.718 ± 0.584 g at 2 × 10^−4^ M, respectively. Bladder contractions in response to cumulative carbachol stimulation at a concentration of 2 × 10^−4^ M were enhanced at 8 weeks of DM (4.07 ± 1.69 g, *p* = 0.028) (Figure 2b). The ratio of contractions at a concentration of 2 × 10^−4^ M carbachol/KCl 100 mM was also significantly higher (1.59 ± 0.28 g, *p* = 0.028) compared with the control (1.07 ± 0.31 g) as determined by Steel's multiple comparison test. However, the dose–response curve showed no difference in bladder ring contraction in each group depending on the concentration of carbachol (control EC_50_ = 2 × 10^−5.75^ M) (Figure 2c). Data in organ bath studies can be viewed in Table S4.

### 3.5 Bladder morphology

**FIGURE 3:**
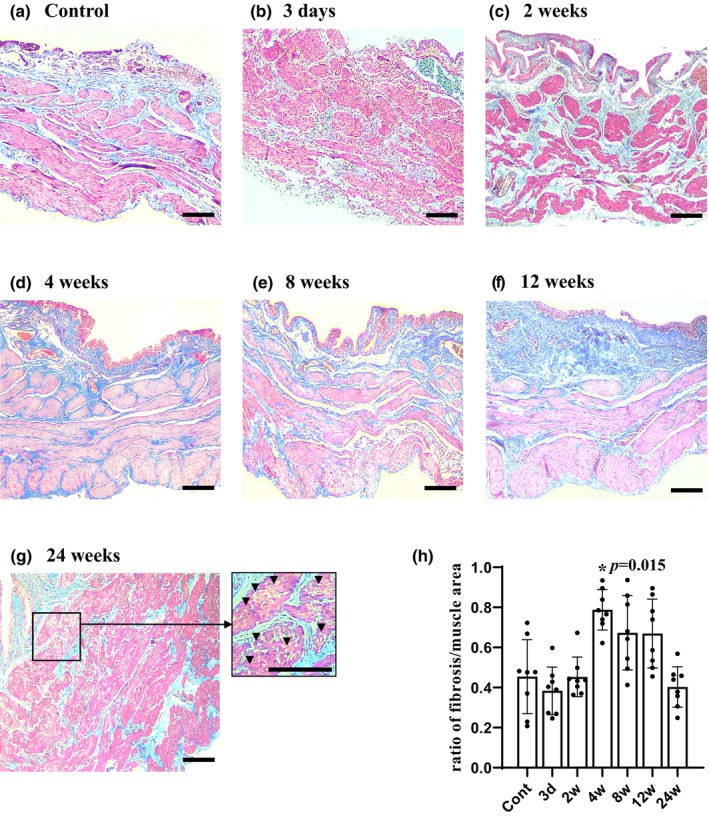
Representative images of Masson's trichrome staining of the bladder in the control (a, *n* = 4) and 3 days and 2, 4, 8, 12, and 24 weeks after diabetes induction (b–g, *n* = 4 each). (h) Increased ratio of the fibrotic/muscle area in the detrusor smooth muscles 4 weeks after diabetes. At 24 weeks, muscle disarrangement or vacuolization (▼) was evident. Data are expressed as the mean ± standard deviation. Statistical significance was determined by Steel's multiple comparison test. *p < 0.05. Scale bars indicate 100 μm.

Masson's trichrome staining showed that the ratio of fibrotic/muscle area at 4 weeks of DM (*n* = 4) was significantly higher (*p* < 0.05) than that in the control group (0.454 ± 0.185, *n* = 4) but returned to the level seen in the control at 24 weeks of DM (0.403 ± 0.101, *n* = 4) by Steel's multiple comparison test (Figure 3). Data in bladder morphology can be viewed in Table S5.

## 4 DISCUSSION

The results of present study indicate that STZ‐induced DM rats showed: (1) urination frequency in the early phase, as evidenced by increased frequencies during the dark period at 3 days of DM compared with the control in the metabolic cage study; (2) dysfunction of decompensated bladder activity in the late phase, as evidenced by prolonged ICI at 8 and 12 weeks of DM and decreased voiding efficiency at 24 weeks of DM in the CMG; (3) preserved bladder contractility, as evidenced by the lack of differences in the dose–response by carbachol stimulation between groups in the organ bath study; (4) turning point of bladder activity changes in the middle phase, as evidenced by increased bladder contractile force by carbachol at 8 weeks of DM in the organ bath study; and (5) changes in bladder morphology, as evidenced by the increased percentage of the fibrotic area of the bladder in the detrusor smooth muscles at 4 weeks of DM.

The findings of the present study show that STZ‐induced diabetes rats showed urination frequency at an early stage. In the present study, the urination frequency during the dark period was significantly higher at 3 days of DM compared with the control in the metabolic cage study. In addition, water intake at 2 weeks and the ratio of total 24 h urine volume/water intake at 3 days, and 4, 8, and 24 weeks increased compared with the control in the metabolic cage study. Therefore, one of main reasons for urination frequency in the early phase might be due to significant increases in fluid intake and urine output by hyperglycemia. The bladder afferents of pelvic nerves carry sensory information through the pontine micturition center of the spinobulbospinal reflex pathway to induce bladder contractions, particularly by Aδ myelinated fibers (Fowler et al., 2008). Since an overactive bladder results in changes in the bladder afferent signaling pathway (Aδ and C fibers) (Cao et al., 2019), urinary frequency may be caused by the degenerative changes in the afferents nerve pathway involving the micturition in the early compensated phase after DM. Daneshgari et al. (Daneshgari, Huang, et al., 2006; Daneshgari, Liu, & Imrey, 2006) asserted that bladder capacity and compliance have been reported to increase in the early phase at 2–8 weeks of STZ‐induced DM. Thus, the other possibility for urination frequency in the early phase might be denervated bladder afferent pathways for ischemia due to overdistension (Daneshgari, Huang, et al., 2006, Daneshgari, Liu, & Imrey, 2006) and inflammation after DM. These changes may have led to the genesis of fibrosis at 4 weeks of DM in the present study. However, in the present study, ICI did not change in the early phase after DM. For calculating bladder compliance, it is necessary to perform a single cystometry as humans (Fraser et al., 2020), however, our method utilized continuous cystometry and DM rats show prominent RUV thus cannot match the formula (fluid volume infused/pressure threshold‐baseline pressure) (Yoshiyama et al., 2008). Instead, our method of calculating VE is valid (Gotoh et al., 2023). VE did not worsen in the early phase. Therefore, this discrepancy wherein ICI was not shortened in the present study might be due to preserved VE in the early phase.

The findings of the present work reveal that the middle phase at 8 weeks after DM might be a turning point from the compensatory phase to the decompensatory phase. Denervated organs are hypersensitive to neurotransmitters (Klee et al., 2019; Yoshida et al., 1982). In a type 2 rat model established using a high‐fat diet and low doses of STZ, increased detrusor contractility in response to the addition of carbachol or adenosine triphosphate was observed in bladder muscle strips (Klee et al., 2019). In the present study, bladder contractions in response to 2 × 10^−4^ M carbachol /KCl were also enhanced at 8 weeks of DM compared with the control in the organ bath studies. These responses are called denervation supersensitivity; thus, bladder afferent activity may change in the middle phase after DM. Denervation supersensitivity is caused by a decrease in nerve endings and an increased sensitivity to neurotransmitters before nerve damage progresses. This phenomenon causes bladder smooth muscle overactivity, which clinically manifests as bladder overactivity with increased urination frequency (Andersson & Wein, 2004). Liu and Daneshgari (2005) also reported that bladder contractions were increased by electrical field stimulation of muscle strips in 9‐week STZ DM rats, which might be consistent with our present findings of hypersensitivity to carbachol at 8 weeks. In diabetic rats, upregulation of M2 (Tong et al., 2002) and M3 (Tong & Cheng, 2002) muscarinic receptors has also been reported. Therefore, in the present study, the hypersensitivity to carbachol in STZ‐induced type I DM may be due to the upregulation of muscarinic receptors. Moreover, a decrease in the autonomic nerves supplying the detrusor muscle has been reported in patients with bladder outlet obstruction (Gosling et al., 1986; Harrison et al., 1987) or idiopathic detrusor instability (Mills et al., 2000). Thus, denervation may occur during the early phase of an overactive bladder. Relatively mild increases in glucose levels may have induced slow degenerative changes in nerves in the middle phase at 8 weeks because body weight was increased in DM groups such as type II DM in the present study. However, a leftward shift of the curve in carbachol dose–response curves at 8 weeks of DM rats was not shown in the present study (as shown in Figure 2c), therefore further studies are required to clarify this point.

The findings of the present study demonstrate that long‐term DM induced decompensatory bladder activity. As for the decompensated state, impaired urine output and increased RUV were observed after DM due to the duration of hyperglycemia (Frimodt‐Møller, 1980). As 1 rat day is equivalent to 1 month in humans (Sengupta, 2013), 24 weeks in the present study represents approximately 14 years of DM duration in humans. ICI was prolonged at 8–12 weeks, and RUV and voiding efficiency increased and decreased, respectively at 24 weeks of DM in the CMG. Consistent with the metabolic cage experiment and CMG in the present study, urination frequency in the early phases at 3 days returned to the control level at 24 weeks of DM, and the prolonged ICI at 8–12 weeks was shortened at 24 weeks, possibly because of the increase in RUV. Analysis of the bladder morphology revealed that the increased percentage of the fibrotic area of the bladder in the detrusor smooth muscle at 4 weeks returned to the control level at 24 weeks, possibly due to bladder muscle hypertrophy and a relative decrease in the fibrotic area, which may be consistent with the lack of changes in bladder weight. However, the maximal contraction pressure and dose–response curve after carbachol treatment did not decrease at 24 weeks of DM. Masuda et al. (2020) showed that diabetic rats exhibited a high contractile response to carbachol at 4–16 weeks, which was reversed at 16 weeks. Moreover, Kendig et al. (2016) reported that in a male type II DM model, particularly ZDF rats, contractility and sensitivity to carbachol and adenosine triphosphate in the smooth muscle strips of the bladder increased at 27 weeks of age. These findings suggest that muscle strength is preserved for a relatively long time, even in a decompensated state. Overall, the decreased urine output and increased RUV in the late phase could be primarily due to the degenerative changes in the nerves involved in micturition after DM. Daneshgari, Liu, and Imrey (2006) reported similar work as long as 20 weeks after DM examining from decompensated to compensated phase. To the best of our knowledge, this study is, to date, the longest‐term to demonstrate the changes of storage and voiding bladder activity in a type I DM model.

Relaxation of urethral smooth muscle/external urethral sphincter innervated by pudendal nerves is crucial for efficient voiding (Fowler et al., 2008). Therefore, urethral activity is another concern related to the time‐dependent effects of LUTD. In the present study, the voiding efficiency decreased, suggesting that urethral activity was impaired at 24 weeks of DM. STZ‐induced diabetic rats have impaired bladder and urethral coordination due to a decrease in nitric oxide production (Yoshimura & Chancellor, 2003) or soluble guanylyl cyclase levels (Gotoh et al., 2021), which induces urethral smooth muscle relaxation by increasing intracellular cyclic guanosine monophosphate levels. The other report showed discoordinated action of external urethral sphincter innervated by pudendal nerves, as evidenced by external urethral sphincter contracts during urethral relaxation by simultaneous recordings of bladder activity and urethral perfusion pressure (Torimoto et al., 2004), and abnormal electromyogram changes (Liu et al., 2008) in STZ‐induced diabetic rats. However, we did not directly measure urethral activity by using urethral perfusion pressure combined with bladder pressure, warranting further studies.

Despite the findings, this study has some limitations. First, we used an STZ‐induced type I DM model. Clinically, type II DM is the most common in humans, and it is difficult to apply our results to the entire DM population. Second, our model has large scattering, especially in females, as females have been reported to be STZ‐resistant (Furman, 2021; Ghasemi & Jeddi, 2023). Third, urethane anesthesia may affect the bladder/urethral function. Fourth, we did not measure direct afferent activity nor did we monitor urethral activity. Finally, aging itself may affect bladder/urethral function. However, in order to reduce the number of animals used in the experiment, we did not examine age‐matched sham controls.

Nonetheless, the results of the present study indicate the time‐dependent progression of diabetic LUDS, focusing on storage and voiding bladder activity, suggesting that it may be useful for treatment options for stopping the progression of decompensated diabetic LUDS.

## 5 CONCLUSIONS

This study demonstrates that diabetic LUTD arises due to damage to bladder activity in the early phase, which progresses to the decompensated phase in the underactive bladder with increased RUV and decreased voiding efficiency; however, bladder contractility is preserved even in the late phase. These mechanisms are helpful when considering the treatment options for diabetic LUTD.

## AUTHOR CONTRIBUTIONS

The contributions of each author are as follows: (1) Conception, experimental design, and investigation: KI, TCK, TO, RK, TA, and MM; (2) Statistical application: KI, TCK, TO, RK, MK, TA, and MM; (3) Critical drafting and revision of the article for important intellectual content: KI, TCK, TO, RK, AA, MK, TA, and MM; and (3) Final approval of the version to be published: KI, TCK, TO, RK, AA, MK, TA, and MM. All contributors who did not meet the criteria for authorship are listed in the Acknowledgements section.

## FUNDING INFORMATION

This work was supported by JSPS KAKENHI (grant numbers 18K17752, 21K11218, 22K17589, 22K11420, and 24K14349).

## CONFLICT OF INTEREST STATEMENT

The authors have no competing interests to declare that are relevant to the content of this article.

## Supporting information


Data S1.

## Data Availability

All relevant data are included in the manuscript and supporting information files.
