# Supplementary material for: Time‐dependent bladder activity changes in streptozotocin‐induced female diabetic rats
Source: Physiol Rep. 2025 Feb 20;13(4):e70220. doi: 10.14814/phy2.70220 (PMC11842456; doi:10.14814/phy2.70220)
Supplement: Supplementary file 1 [file PHY2-13-e70220-s001.docx]

Table S1. general characteristics

**control**	N1	N2	N3	N4	N5
body weight	224	227	217	217	218
bladder weight	0.113	0.131	0.1012	0.157	0.142
bladder/body(10^3)	0.5044642857	0.577092511	0.466359447	0.7302325581	0.65137615
Blood glucose (mg/dL)	204	196	151	214	169

**DM 3days**	N1	N2	N3	N4	N5
body weight	268	260	194	194	187
bladder weight	0.1166	0.142	0.0996	0.1013	0.1128
bladder/body(10^3)	0.4350746269	0.5461538462	0.5134020619	0.5221649485	0.6032085561
Blood glucose (mg/dL)	287	410	315	613	834

**DM 2weeks**	N1	N2	N3	N4	N5
body weight	235	245	253	218	249
bladder weight	0.159	0.176	0.138	0.1728	0.162
bladder/body(10^3)	0.6765957447	0.7183673469	0.5454545455	0.7926605505	0.6506024096
Blood glucose (mg/dL)	900	452	461	897	900

**DM 4weeks**	N1	N2	N3	N4	N5
body weight	291	324	260	309	278
bladder weight	0.258	0.24	0.143	0.203	0.148
bladder/body(10^3)	0.8865979381	0.7407407407	0.55	0.6569579288	0.5323741007
Blood glucose (mg/dL)	688	394	305	722	324

**DM 8weeks**	N1	N2	N3	N4	N5
body weight	289	317	273	291	307
bladder weight	0.244	0.144	0.142	0.143	0.180
bladder/body(10^3)	0.8442906574	0.4542586751	0.5201465201	0.4914089347	0.5863192182
Blood glucose (mg/dL)	461	556	355	706	386

**DM 12weeks**	N1	N2	N3	N4	N5
body weight	315	319	315	304	309
bladder weight	0.52	0.2802	0.1691	0.259	0.308
bladder/body(10^3)	1.6507936508	0.878369906	0.5368253968	0.8519736842	0.99676375
Blood glucose (mg/dL)	679	525	900	545	739

**DM 24weeks**	N1	N2	N3	N4	N5
body weight	334	259	325	307	286
bladder weight	0.1248	0.347	0.362	0.315	0.211
bladder/body(10^3)	0.3736526946	1.3397683398	1.1138461538	1.0260586319	0.7377622378
Blood glucose (mg/dL)	701	665	511	900	702

**DM 2weeks**	N1	N2	N3	N4	N5	N6
watar intake (g)	55.05	53.91	70.64	48.04	57.18	45.97
urination frequency (light)	10	12	13	9	10	7
urination frequency (dark)	27	16	19	12	17	12
urination frequency (total)	37	28	32	21	27	19
urinary volume (ml/day)	33.71	18.1	31.19	25.9	26.03	15.91
urinary volume (ml/time)	0.911	0.646	0.975	1.233	0.964	0.837
Urinary vol./ Water intake	0.612	0.336	0.442	0.539	0.455	0.346

**DM 4weeks**	N1	N2	N3	N4	N5	N6
watar intake (g)	37.81	40.23	35.03	64.53	34.16	30.48
urination frequency (light)	9	10	11	8	7	11
urination frequency (dark)	11	18	12	41	19	14
urination frequency (total)	20	28	23	49	26	25
urinary volume (ml/day)	21.54	27.19	23.11	75.44	21.2	22.14
urinary volume (ml/time)	1.077	0.971	1.005	1.540	0.815	0.886
Urinary vol./ Water intake	0.570	0.676	0.660	1.169	0.621	0.726

**DM 8weeks**	N1	N2	N3	N4	N5	N6	N7	N8
watar intake (g)	75.76	56.085	16.356	26.979	18.331	42.13	28.84	43.37
urination frequency (light)	5	4	9	4	5	10	12	14
urination frequency (dark)	32	24	6	11	7	22	9	18
urination frequency (total)	37	28	15	15	12	32	21	32
urinary volume (ml/day)	75.22	62.63	16.57	20.47	11.24	28.61	13.85	27.56
urinary volume (ml/time)	2.033	2.237	1.105	1.365	0.937	0.894	0.660	0.861
Urinary vol./ Water intake	0.993	1.117	1.013	0.759	0.613	0.679	0.480	0.635

**DM 12weeks**	N1	N2	N3	N4	N5	N6	N7	N8
watar intake (g)	56.01	61.41	53.65	58.04	78.33	18.56	113.19	26.17
urination frequency (light)	11	8	5	6	11	4	8	7
urination frequency (dark)	18	23	21	14	28	9	39	15
urination frequency (total)	29	31	26	20	39	13	47	22
urinary volume (ml/day)	28.78	27.46	27	17.22	47	11.07	118.7	18.77
urinary volume (ml/time)	0.992	0.886	1.038	0.861	1.205	0.852	2.526	0.853
Urinary vol./ Water intake	0.5138368149	0.4471584433	0.5032618826	0.2966919366	0.600025533	0.5964439655	1.0486792119	0.7172334734

**DM 24weeks**	N1	N2	N3	N4	N5	N6
watar intake (g)	37.647	22.784	3.188	25.105	1.035	156.025
urination frequency (light)	10	9	5	9	12	15
urination frequency (dark)	14	9	6	10	15	31
urination frequency (total)	24	18	11	19	27	46
urinary volume (ml/day)	18.99	13.81	11.87	15.39	53.56	142.43
urinary volume (ml/time)	0.791	0.767	1.079	0.810	1.984	3.096
Urinary vol./ Water intake	0.5044226632	0.6061271067	3.7233375157	0.6130252938	51.748792271	0.9128665278

voiding volume (mL)	0.26	0.71	0.20	0.28	0.29	0.15
residual volume (mL)	0.16	0.23	0.00	0.00	0.2	0.27
voiding efficiency	0.619	0.755	1.000	1.000	0.591837	0.3571428571

**DM 4weeks**	N1	N2	N3	N4	N5	N6
basal pressure (cmH2O)	7.580	5.129	9.992	4.876	8.718	9.848
threshold pressure ((cmH2O)	8.217	7.911	11.300	7.994	13.950	12.770
Max reflex contraction oressure (cmH2O)	15.007	31.212	30.256	28.528	28.338	23.802
intercontraction interval (sec)	270.3	525.7	161.9	445.0	926.4	369.6
non-voiding contractions	0	1	1	0	3	0
duration of bladder contraction (sec)	36.7	24.0	28.7	32.0	60.0	26.0
voiding volume (mL)	0.13	0.49	0.62	0.40	0.86	0.51
residual volume (mL)	0.10	0.15	0.08	0.15	0.06	0.00
voiding efficiency	0.556	0.766	0.886	0.727	0.935	1.000

**DM 8weeks**	N1	N2	N3	N4	N5	N6
basal pressure (cmH2O)	3.890	3.462	16.675	5.729	5.128	7.799
threshold pressure ((cmH2O)	7.026	5.448	20.516	6.900	6.647	8.657
Max reflex contraction oressure (cmH2O)	30.857	23.821	40.222	25.170	23.525	18.541
intercontraction interval (sec)	263.0	597.7	393.3	369.0	474.3	665.8
non-voiding contractions	0	4	2	2	0	0
duration of bladder contraction (sec)	31.3	22.0	28.0	26.0	26.8	24.0
voiding volume (mL)	0.52	0.40	0.35	0.30	0.30	0.20
residual volume (mL)	1.37	0.08	0.75	0.05	0.24	0.05
voiding efficiency	0.275	0.833	0.318	0.857	0.556	0.800

**DM 12weeks**	N1	N2	N3	N4	N5	N6	N7	N8
basal pressure (cmH2O)	8.131	10.907	4.975	0.146	8.672	6.448	7.865	6.485
threshold pressure ((cmH2O)	13.197	21.166	6.583	2.327	10.483	7.027	11.595	11.950
Max reflex contraction oressure (cmH2O)	42.258	33.896	17.677	26.300	30.473	22.774	37.758	48.832
intercontraction interval (sec)	569.3	688.0	818.5	897.6	603.1	342.4	214.7	1254.0
non-voiding contractions	2	2	0	0	0	0	1	0
duration of bladder contraction (sec)	35.3	19.3	30.0	24.0	23.3	26.0	27.0	34.3
voiding volume (mL)	0.50	0.40	0.65	0.75	0.45	0.40	0.30	0.60
residual volume (mL)	0.10	0.10	0.10	0.10	0.05	0.10	4.40	1.00
voiding efficiency	0.833	0.800	0.867	0.882	0.900	0.800	0.064	0.375

**DM 24weeks**	N1	N2	N3	N4	N5	N6
basal pressure (cmH2O)	3.847	4.653	4.132	7.605	4.559	5.560
threshold pressure ((cmH2O)	14.283	7.207	6.170	8.053	7.428	10.428
Max reflex contraction oressure (cmH2O)	19.618	18.536	18.609	26.082	20.211	21.211
intercontraction interval (sec)	260.3	221.3	234.0	1649.0	342.7	839.3
non-voiding contractions	2	1	0	0	0	1
duration of bladder contraction (sec)	36.0	47.7	36.7	56.0	36.7	51.3
voiding volume (mL)	0.14	0.41	0.21	0	0.32	0.22
residual volume (mL)	0.45	1.8	0.34	2.24	1.00	0.9
voiding efficiency	0.237	0.186	0.382	0.000	0.242	0.196

carbachol 2x10^-6 (cmH_2O)	0.0066	0.0161	0.0445	0	0.8854
carbachol 2x10^-5 (cmH_2O)	0.9866	1.0761	0.8445	0.0333	2.1999
carbachol 2x10^-4 (cmH_2O)	1.5866	1.6861	1.2538	0.4522	2.4054
KCL	1.5518	1.6912	0.997	0.639	1.5654

**DM 2weeks**	bath 1	bath 2	bath 3	bath 4	bath 5	bath 6	bath 7
carbachol 2x10^-8 (cmH_2O)	0	0	0	0	0.0137	0	N/A
carbachol 2x10^-7 (cmH_2O)	0.0334	0	0.0022	0	0.1727	0	N/A
carbachol 2x10^-6 (cmH_2O)	0.9134	0.1386	0.0022	1.1236	1.2427	0.3835	N/A
carbachol 2x10^-5 (cmH_2O)	1.0534	0.4486	0.2022	4.3936	3.4027	1.5248	N/A
carbachol 2x10^-4 (cmH_2O)	0.9134	0.3386	0.5222	4.8036	5.2527	1.9313	N/A
KCL	0.2081	0.0899	0.725	4.2226	3.4111	2.0819	1.169

**DM 4weeks**	bath 1	bath 2	bath 3	bath 4	bath 5	bath 6
carbachol 2x10^-8 (cmH_2O)	0	0	0	0	0	0
carbachol 2x10^-7 (cmH_2O)	0	0.0077	0	0.0967	0.0033	0
carbachol 2x10^-6 (cmH_2O)	0.0888	0.4877	0.1216	0.6067	0.6633	0.3263
carbachol 2x10^-5 (cmH_2O)	1.6888	1.3977	0.6716	2.1367	1.3133	1.1563
carbachol 2x10^-4 (cmH_2O)	2.4188	1.5877	0.7616	2.2867	1.3333	1.2863
KCL	1.4639	1.2703	0.3873	1.2286	1.5312	0.9871

**DM 8weeks**	bath 1	bath 2	bath 3	bath 4	bath 5	bath 6	bath 7	bath 8
carbachol 2x10^-8 (cmH_2O)	0.0229	0.0103	0	0.0171	0	0.0367	0	0
carbachol 2x10^-7 (cmH_2O)	0.3693	0.3703	0.0094	0.0402	0.3165	0.1794	0.023	0.0696
carbachol 2x10^-6 (cmH_2O)	1.9893	1.9803	0.5686	1.0082	1.4588	0.6773	0.5054	0.9124
carbachol 2x10^-5 (cmH_2O)	6.1293	4.3603	2.4105	3.4781	3.3302	2.9142	1.2058	2.4908
carbachol 2x10^-4 (cmH_2O)	7.2393	5.8503	3.036	4.3577	3.6464	3.2206	2.1733	3.0351
KCL	4.8811349668	3.8494	2.0996	2.9652	1.8794	2.1506	1.6954	1.432

**DM 12weeks**	bath 1	bath 2	bath 3	bath 4	bath 5	bath 6
carbachol 2x10^-8 (cmH_2O)	0	0	0.0170430789	0.0202196596	0	N/A
carbachol 2x10^-7 (cmH_2O)	0.3871	0.0031	0.0210548764	0.1327196596	0	N/A
carbachol 2x10^-6 (cmH_2O)	2.1971	0.3124	0.6107891026	0.6434196596	0.0945	N/A
carbachol 2x10^-5 (cmH_2O)	3.6471	0.9846	1.6017030746	1.8464196596	0.6923	N/A
carbachol 2x10^-4 (cmH_2O)	4.1771	1.5579	1.9226	1.8638196596	0.9042	N/A
KCL	2.607	2.0642	1.1893	1.2537	1.9601	1.8065

**DM 24weeks**	bath 1	bath 2	bath 3	bath 4	bath 5	bath 6
carbachol 2x10^-8 (cmH_2O)	0.0825	0	0.0246	0	0	0
carbachol 2x10^-7 (cmH_2O)	0.1685	0.3687	0.1585	0.0343	0.1638	0.1931
carbachol 2x10^-6 (cmH_2O)	0.5518	0.7943	0.6102	0.5699	0.8017	1.1756
carbachol 2x10^-5 (cmH_2O)	0.8491	1.0936	2.3633	2.088	2.5449	2.3326
carbachol 2x10^-4 (cmH_2O)	0.9327	1.1708	2.7193	2.8785	3.1577	3.1558
KCL	0.3972	0.5806	2.668	1.707	1.8582	1.8829

Table S5. bladder morphology

**control**	muscle area	collagen area
1	882873	420848
2	945016	216955
3	867917	180275
4	1000224	481776
5	1063505	506705
6	910730	337255
7	788104	527094
8	836659	604949

**DM3d**	muscle area	collagen area
1	1187320	319274
2	953721	260348
3	879772	354985
4	872972	401332
5	1250734	486810
6	1375511	339253
7	1087612	466625
8	828811	495706

**DM2w**	muscle area	collagen area
1	1096015	399095
2	1096173	486424
3	962168	420560
4	844056	420982
5	884598	383032
6	874202	354157
7	773558	520570
8	973245	360712

**DM4w**	muscle area	collagen area
1	707882	634076
2	749829	466170
3	788263	582777
4	707721	555983
5	1032983	743237
6	931200	713462
7	754191	705080
8	489061	410364

**DM8w**	muscle area	collagen area
1	981584	405292
2	790796	678964
3	1048608	674013
4	991645	675872
5	1041509	566921
6	1006403	497876
7	844861	790840
8	1082622	881592

**DM12w**	muscle area	collagen area
1	1037186	607672
2	1192892	543221
3	1013672	791603
4	1158313	577335
5	1092121	977611
6	1031462	892009
7	899096	480949
8	975224	721221

**DM24w**	muscle area	collagen area
1	1146951	642246.3414[illegible]
2	1211439	771166.8693[illegible]
3	1151050	675148.8014[illegible]
4	994792	738276.3884[illegible]
5	1242833	784938.6412[illegible]
6	1165643	592462.3343[illegible]
7	1433923	903749.6062
8	1242820	983938.4040
